# Global Changes in Gene Expression of Barrett's Esophagus Compared to Normal Squamous Esophagus and Gastric Cardia Tissues

**DOI:** 10.1371/journal.pone.0093219

**Published:** 2014-04-08

**Authors:** Paula L. Hyland, Nan Hu, Melissa Rotunno, Hua Su, Chaoyu Wang, Lemin Wang, Ruth M. Pfeiffer, Barbara Gherman, Carol Giffen, Cathy Dykes, Sanford M. Dawsey, Christian C. Abnet, Kathryn M. Johnson, Ruben D. Acosta, Patrick E. Young, Brooks D. Cash, Philip R. Taylor

**Affiliations:** 1 Division of Cancer Epidemiology and Genetics, National Cancer Institute, National Institutes of Health, Bethesda, Maryland, United States of America; 2 Cancer Prevention Fellowship Program, Division of Cancer Prevention, National Cancer Institute, National Institutes of Health, Bethesda, Maryland, United States of America; 3 Westat, Rockville, Maryland, United States of America; 4 Information Management Services, Inc, Silver Spring, Maryland, United States of America; 5 Walter Reed National Military Medical Center, Bethesda, Maryland, United States of America; Peter MacCallum Cancer Centre, Australia

## Abstract

**Background:**

Barrett's esophagus (BE) is a metaplastic precursor lesion of esophageal adenocarcinoma (EA), the most rapidly increasing cancer in western societies. While the prevalence of BE is increasing, the vast majority of EA occurs in patients with undiagnosed BE. Thus, we sought to identify genes that are altered in BE compared to the normal mucosa of the esophagus, and which may be potential biomarkers for the development or diagnosis of BE.

**Design:**

We performed gene expression analysis using HG-U133A Affymetrix chips on fresh frozen tissue samples of Barrett's metaplasia and matched normal mucosa from squamous esophagus (NE) and gastric cardia (NC) in 40 BE patients.

**Results:**

Using a cut off of 2-fold and *P*<1.12E-06 (0.05 with Bonferroni correction), we identified 1324 differentially-expressed genes comparing BE vs NE and 649 differentially-expressed genes comparing BE vs NC. Except for individual genes such as the *SOXs* and *PROM1* that were dysregulated only in BE vs NE, we found a subset of genes (n = 205) whose expression was significantly altered in both BE vs NE and BE vs NC. These genes were overrepresented in different pathways, including TGF-β and Notch.

**Conclusion:**

Our findings provide additional data on the global transcriptome in BE tissues compared to matched NE and NC tissues which should promote further understanding of the functions and regulatory mechanisms of genes involved in BE development, as well as insight into novel genes that may be useful as potential biomarkers for the diagnosis of BE in the future.

## Introduction

Esophageal cancer is the sixth leading cause of cancer-related deaths worldwide and exhibits dramatic geographic differences in distribution in incidence and histological subtype [1]. Over the last 35 years in the United States (USA), the incidence of esophageal adenocarcinoma (EA) has increased from 0.4 to more than 3 per 100,000 person-years, a 650% increase [2]–[4]. When gastroesophageal reflux disease (GERD) induces inflammation in the normal esophageal (NE) squamous epithelium, the damaged squamous cells are usually replaced by regeneration of more squamous cells. In some individuals, however, the reflux-damaged NE heals through a metaplastic process in which intestinal-type columnar cells (specialized intestinal metaplasia, SIM) replaces the reflux-damaged squamous epithelium [5]. This metaplasia results in Barrett's esophagus (BE), which is the recognized precursor lesion to EA [6]–[8], and increases the risk of EA by 11 times compared to that of the general population [9].

In addition to male sex and Caucasian race, the most well-documented risk factors for BE include increasing age, cigarette use, obesity, and a lack of *Helicobacter pylori (H. pylori)* infection [10], [11]. However, the strongest risk factor for BE is GERD [11], which is also a primary risk factor for EA [12]. A population-based study from Sweden [13] and a computer simulation using the Surveillance Epidemiology and End Results (SEER) data [14], suggest that 1.5–5.6% of the general population of western societies have BE. However, recent data indicate that the incidence of BE has increased and is rising [15], [16]. Coleman et al. reported that average annual incidence rate of BE increased by 159% in the UK from 1993–2005, with a marked increase in individuals over 60 years, and particularly amongst males under 40 years [16]. Although, less than 5% of patients with BE will go on to develop EA, it is generally accepted that most persons with BE are undiagnosed and the vast majority of EA occurs in patients with undiagnosed BE. For example, in the US a high prevalence of BE (7–25% for segments of any length) was reported in asymptomatic patients who agreed to have an upper gastrointestinal endoscopy screening when attending for colonoscopy [17]. Furthermore, the most commonly used means of early detection of EA is endoscopic examination [18]; however at a population level this approach for screening is neither feasible nor cost-effective.

Previous biochemical studies have shown that BE has features in common with gastric mucosa, including mucus secretory capacity, mucus granules, and expression of columnar cell cytokeratins [19], but it also shares features with squamous esophageal cells, its presumed precursor, including expression of squamous cell cytokeratins [19], [20]. In an attempt to advance our understanding of the etiology of BE and its progression to EA at the molecular level, as well as to identify potential gene targets for evaluation as diagnostic markers, numerous studies have reported on the differential expression of genes between BE and EA tissues and also between BE and normal squamous esophagus (NE) tissues [21]–[26]. However, only two studies to date have included a comparison of matched BE, NE, and normal gastric cardia (NC) tissues from the same patient. In 2002, Barrett et al. [21] compared the gene expression profiles from pooled BE, NE, and NC tissues using the HU6800 microarray, while a subsequent study [22] compared transciptomes from each of these tissues using serial analysis of gene expression (SAGE). Both of these techniques for quantitating gene expression have their limitations; the HU6800 microarray is specific, but contains probes for only 7000 genes in the genome, while SAGE, which is highly specific and very reproducible for abundant transcripts, can be prone to sequencing errors.

In this study we used Affymetrix HG-U133A microarrays on fresh frozen matched BE, NE, and NC tissues from 40 BE patients to evaluate differentially-expressed genes between these tissues. Specifically, we compared gene expression between BE and NE tissues and between the BE and NC tissues and identified major genes that were differentially expressed, using stringent criteria for gene selection (fold-change >2.0 and *P*<1.12E-06). We then performed Gene Ontology classification and pathway-based analyses to identify functional groups, pathways, and key regulators among the identified genes.

## Methods

### Ethics statement, study population and tissue collection

This study was approved by the Institutional Review Boards of the Walter Reed National Military Medical Center (WRNMMC) and the National Cancer Institute (NCI), USA. BE patients were recruited as part of the Barrett's Esophagus Early Detection Study (BEEDS), a case-control study conducted among patients presenting to the Gastroenterology Department at the WRNMMC in Bethesda, Maryland, USA. After obtaining written informed consent, patients were interviewed to obtain information on demographic, lifestyle, and clinical factors, other clinical data were retrieved from the medical record, a blood sample was taken, and tissue samples were obtained during endoscopy.

Clinical data were collected for each patient by an attending nurse and a GERD questionnaire (modified from Manterola *et al.* [27]) was administered to all participants. Esophagogastroduodenoscopy (EGD) was performed on all patients, using a GIF-Q180 gastroscope (Olympus). During endoscopy, a gastroenterologist used disposable forceps to obtain multiple mucosal biopsies of normal gastric cardia (NC; within one cm distal to the gastroesophageal junction or the top of the gastric folds), Barrett's esophagus (BE; four quadrant biopsies in accord with surveillance guidelines; if present), and normal esophageal (NE) squamous tissue (at 30 cm from the gums) from the same patient. The gastroenterologist first obtained a clinical biopsy that was placed in formalin for use in determining histological diagnosis. A second research biopsy was then taken as close as visually/endoscopically possible to the clinical biopsy. The research biopsy was snap frozen in liquid nitrogen and stored at −130°C until required for RNA extraction.

### RNA preparation and microarray methods

RNA was extracted from whole frozen tissue biopsies using Trizol (Life Technologies) according to manufacturers' instructions; RNA purity and quantity were determined using an RNA 6000 Labchip/Agilent 2100 Bioanalyzer (Agilent Technology, Inc.). Each microarray experiment was carried out using 15 μg of total RNA and probes were prepared as described previously [28]. Twenty micrograms of biotinylated cDNA were applied to each GeneChip Human Genome U133A 2.0 hybridization array and all tissue RNAs from the same BE patient were processed on separate arrays in the same batch. After hybridization at 45°C overnight, arrays were developed with phycoerythrin-conjugated streptavidin by using a fluidics station (Genechip Fluidics Station 450) and scanned (Genechip Scanner 3000) to obtain quantitative gene expression levels [28]. Paired BE and normal tissue specimens from each patient were processed simultaneously throughout the RNA extractions and hybridizations.

### Validation of microarray results by real-time RT-PCR

A total of nine genes (Table S6) with a >2-fold expression difference either in BE vs NE and/or BE vs NC were selected for technical validation and independent replication. We carried out a technical validation of two random genes (*CD36* and *SLC6A14*) as well as an independent validation of seven other genes (*ABP1*, *ATP2C2*, *CALML4*, *HOXB7 KRT7*, *MSLN*, and *TFF3*), six of which have previously been reported to be differentially expressed in BE and implicated in its development. Increased expression of the remaining gene *MSLN* has been reported in other cancers and was significantly upregulated in both comparison groups from our array data. For each gene target, real-time quantitative PCR (qPCR) was carried out using cDNA from BE-, NE-, and NC-matched RNAs. Amplification conditions yielded efficiencies >90% and linear regression coefficients >0.990 for all assays which were carried out as previously described in http://docs.appliedbiosystems.com/search. All reactions were performed in triplicate using commercially available kits (Applied Biosystems Inc.). *GAPDH* was used as the internal control and PCRs were carried out on an ABI Prism 7000 Sequence Detection System. The average *C*_T_ was calculated for each gene evaluated and *GAPDH*, and the Δ*C*_T_ was determined as the mean of the triplicate *C*_T_ values for the evaluated gene minus the mean of the triplicate *C*_T_ values for *GAPDH* [28]. The N-fold differential expression of the evaluated gene for a BE sample compared with its normal epithelial counterpart was expressed as 2^−ΔΔ*C*T^ (formula ΔΔ*C*_T_ = Δ*C*_T_ of BE−Δ*C*_T_ of its normal epithelial counterpart), which represents the fold change in the target gene expression in BE normalized to an internal control gene (*GAPDH*) and relative to the normal comparator tissues (NE and NC epithelial tissues, respectively).

### Statistical analyses

Statistical analyses were carried out using R program language (http://www.r-project.org/). Gene expression data were processed and normalized using the Bioconductor Affy package, based on the Robust Multichip Average (RMA) method for single-channel Affymetrix chips. The GEO accession number for this array set is GSE39491. All 22,277 probe sets based on the RMA summary measures were used in class comparison analyses. For analyses including paired tissues (BE vs NE and BE vs NC from the same subjects), a linear mixed effects model was used to account for intra-person correlation. For comparative purposes at the individual probe level, we focused on gene probes with *P*-value <1.12E-06 (0.05/44,554 probes, Bonferroni corrected two-sided) and fold-change ≥2. Fold-change (fc) was defined as 2β, where log2 expression = α+β×metaplasia status. Principal component analysis (PCA) was conducted to explore global differences in gene expression profiles.

### Ontology group classification and molecular pathway construction using Pathway Studio 9.0

Gene ontology (GO) [29] was used for functional classifications of genes significantly differentially expressed in both BE vs NE and BE vs NC comparative sets, regardless of direction. We further analyzed this gene set using Pathway Studio software (version 9.0) (Ariadne Genomic, Rockville, MD) (http://www.ariadnegenomics.com/products/pathway-studio/) and the Fisher's exact test. Pathway Studio 9.0 is a text-mining tool that detects relationships among genes, proteins, cell processes, and diseases as recorded in the PubMed database. Pathway Studio 9.0 constructs common regulatory networks by searching the Medscan Database for reported interactions. These analyses allowed us to identify signaling pathways as well as potential regulatory transcription factors and nuclear receptors enriched in our data set.

## Results

### Characteristics of BEED study population

We analyzed 120 tissue samples (BE, NE, and NC) from 40 BE patients. A single pathologist expert in gastrointestinal pathology confirmed all histologic diagnoses of BE from paired adjacent biopsies that were formalin fixed, paraffin-embedded, and H&E stained. BE cases were predominantly male (74%), overweight (median BMI 27.9), and had a mean age of 54.5 years. In addition, alcohol drinking (85%) and smoking (58%) were common amongst BE patients (Table 1). Eighty three percent of BE patients reported having symptoms of GERD (Table 1). Eighty-eight percent (35/40) of BE patients were taking acid suppressants at the time of endoscopic biopsy. Detailed demographic and risk factor information for BE cases are shown in Table S1. Also, endoscopy findings together with pathology findings for BE, NE, and NC biopsies from BE cases (N = 40 cases) are shown in Tables S2 and S3. BE biopsies in cases were all non-dysplastic except for one with high-grade dysplasia and a second that was indeterminate for dysplasia.

**Table 1:** Characteristics of study population.

Covariate		BE patients (n = 40)
**Sex**	male	78%
**Age years**	Year (SD)	54.6 (11.1)
**Tobacco use**	% yes	58%
**Alcohol use**	% yes	85%
**GERD**	% yes	83%
**BMI**		
Mean (SD)		28.5 (4.9)
Median		27.9
Range		15.1–38.0
**The C (Prague) extent of BE epithelium**		
% ≥2 cm		53%
% <2 cm		45%

Persons scoring ≥3 on a scale of 0–13 on the gastroesophageal reflux disease (GERD) questionnaire were considered to have GERD. Tobacco use refers to smoked cigarettes: Yes>6 months ever smoked, No<6 months ever smoked. Alcohol use refers to ever drank alcohol: No/Yes (where No<monthly ever drank over adult life). For the C (Prague) extent of BE epithelium, one subject was unknown. *Abbreviations*: Body mass index, BMI; gastroesophageal reflux disease, GERD.

### Microarray experimental quality control

In the present study, we used the HG_U133A 2.0 hybridization array which contains 22,227 probes sets representing 14,500 genes with multiple probe sets for the same genes (Affymetrix). We assayed hybridization quality by using the Affymetrix GCOS software. The average MAS5 Present call of the 129 HG_U133A chips from the 40 BE patients was 49.9% (range 34.8%–61.8%). The following Affymetrix quality assessment metrics were carried out for all chips: scaling factors; 3′ to 5′ ratio in Beta-Actin and GAPDH; RNA degradation; relative log expression plot; normalized unscaled standard error plot; and chip pseudo-images based on a probe level model (PLM) fit. All chips passed the quality check tests and were used in these analyses. The samples were processed and normalized with the Robust Multichip Average (RMA) method, including background adjustment, quantile normalization, and median polish summarization. No data filtering was applied and all probe sets based on RMA summary measures were used in the analyses.

### Global gene expression signatures

**Figure 1:**
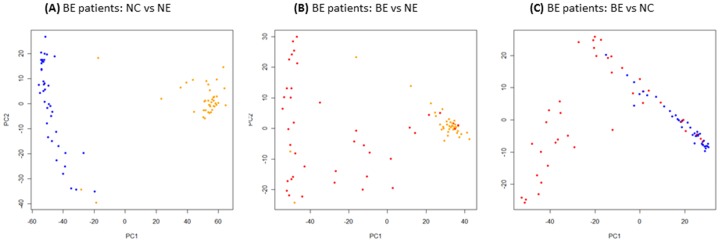
PCA analyses of differentially-expressed probes (≥2 fold change in expression and *P*<1.12E-06) between matched BE, NE, and NC tissues from BE patients. PCA was applied to each set of differentially-expressed probes to reduce the dimensionality of the microarray data with respect to individual samples. Phenotypic subgroups or tissues (BE, NE, and NC) can be differentiated from each other in BE patients, although there is some mixing of the phenotypes, particularly between BE and NC, which are more similar in terms of gene expression profiles. Color key: Blue = NC, Yellow = NE, Red = BE.

**Figure 2:**
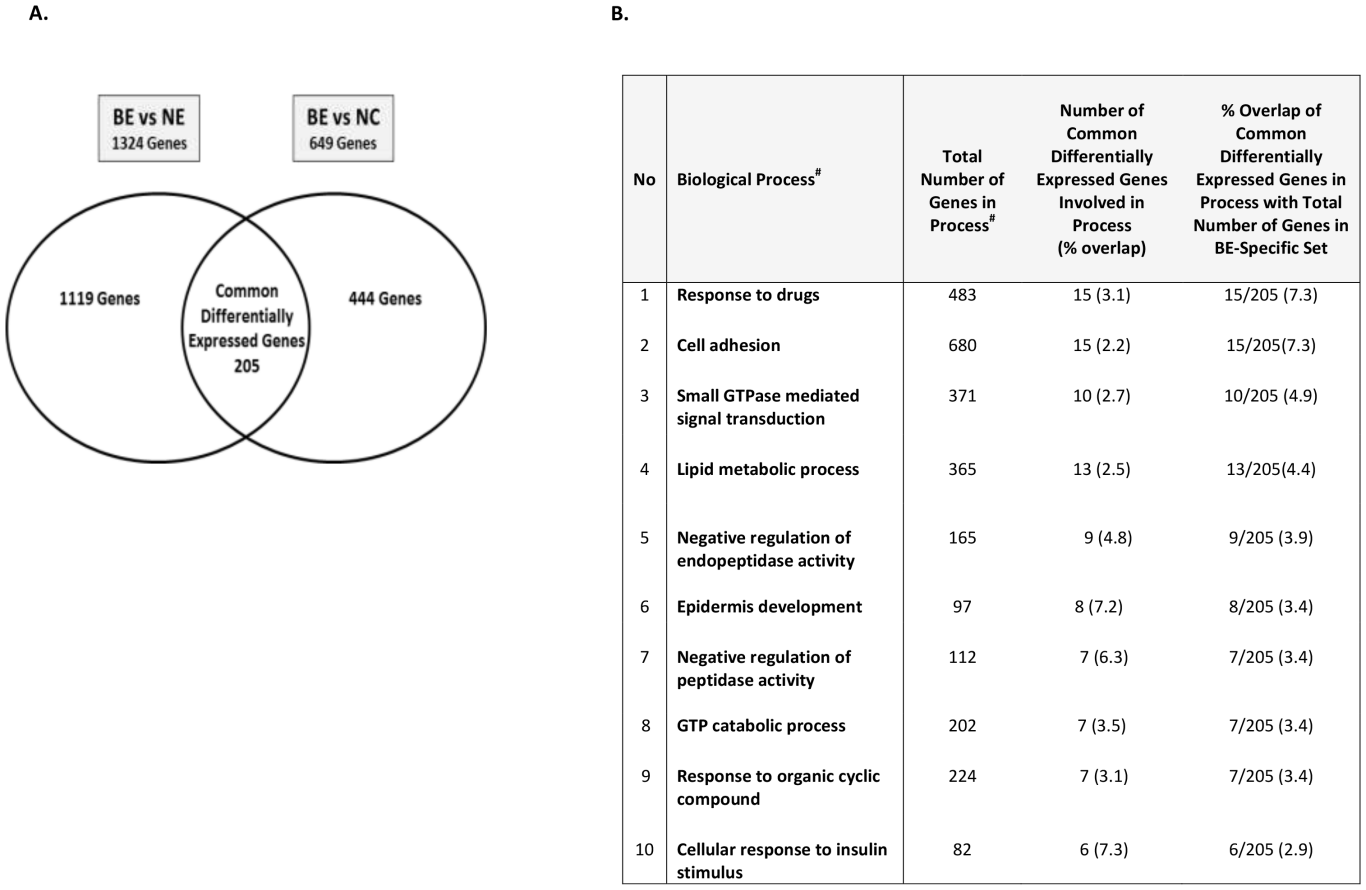
Paired analyses of significant differential gene expression (≥2 fc and *P*<1.12E-06) in BE vs NE and BE vs NC and enrichment in known biological processes. **A.** The left Venn diagram represents the total number of genes with significant differential expression between BE and NE (≥2 fc and *P*<1.12E-06), whereas the right Venn diagram represents the total number with genes significant differential expression between BE and NC (≥2 fc and *P*<1.12E-06). The overlap between the two differentially-expressed sets contained 205 genes used for functional classification and pathway-based analyses. **B.** Enrichment of the 205 genes in known biological processes (GO, Ariadne Genomics, MD). The columns entitled: ‘Total no of genes in process’ and ‘Number of genes involved in process (% overlap)’ refer to the total number of genes currently known in each process according to the database (Pathway Studio 9.00), and the % overlap of the 205 genes with the total gene number in each process, respectively. Enrichment of the 205 genes identified in each process relative to the whole gene set is also shown. The largest proportion of genes (7.3%) out of the 205 genes were involved in response to drugs and cell adhesion; however, relative to the overall number of genes in the identified process, cellular response to insulin showed the greatest enrichment.

Gene probes showed significantly different expression levels among the three tissue types and for each of the comparative analyses (i.e., BE vs NE, BE vs NC). Using a 2 fc cutoff and a Bonferroni correction threshold of 1.12E-06 (0.05/44,554 probe comparisons), a total of 2427 gene probes showed significant differential expression between BE and NE, and BE and NC tissue types, of which, 1645 (967 upregulated and 678 downregulated probes) were between BE and NE tissues, and 782 (491 upregulated and 290 downregulated probes) were between BE and NC tissues (Tables S4 and S5, respectively). PCA analyses of the differentially-expressed probe sets resulted in separation of the samples into their respective tissue type groups. Separation appeared greater for differentially-expressed probes in the BE vs NE comparison than for BE vs NC (Figure 1). Compared to BE vs NE, BE vs NC showed fewer differentially-expressed probes (Table S5); these probes corresponded to 649 differentially-expressed genes (408 upregulated and 241 downregulated) (Figure 2). In contrast, the 1645 differentially-expressed probes identified between BE vs NE tissues corresponded to 1324 genes or gene regions (785 upregulated and 539 downregulated). Summaries showing the top 50 differentially-expressed genes in BE vs NE and BE vs NC tissues are listed in Tables 2 and 3, respectively. Sensitivity analyses that compared these results with results that excluded the single case diagnosed with high-grade dysplasia showed virtually identical results (data not shown).

**Table 2:** Summary showing top 50 differentially expressed genes in BE vs NE (the complete list is reported in Table S4).

No	Gene Symbol	*Gene Name*	*P-value*	Fold Change	Chromosomal Location
1	*MUC5AC^a^*	*Mucin 5AC*	4.68E-16	69.26	11p15.5
2	*CA2*	*Carbonic anhydrase II*	5.11E-16	13.33	8q22
3	*CLDN18^a^*	*Claudin 18*	1.52E-15	53.75	3q22.3
4	*GKN1*	*Gastrokine 1*	2.72E-15	33.65	2p13.3
5	*BMP2*	*bone morphogenetic protein 2*	3.41E-15	6.78	20p12
6	*SULT1C2^b^*	*Sulfotransferase family, cytosolic, 1C, member 2*	4.86E-15	23.93	2q12.3
7	*FER1L4*	*FER1L4 fer-1-like 4 pseudogene*	5.28E-15	8.33	20q11.22
8	*TFF2*	*Trefoil factor 2*	5.47E-15	37.26	21q22.3
9	*PGC*	*Pepsinogen C*	7.01E-15	17.53	6p21.1
10	*POU2AF1*	*POU class 2 associating factor 1*	8.33E-15	15.10	11q23.1
11	*CAPN9^a^*	*Calpain 9*	8.86E-15	13.59	1q42.11-q42.3
12	*CD1A*	*CD1a molecule*	9.42E-15	0.28	1q22-q23
13	*INSR*	*Insulin receptor*	1.03E-14	4.61	19p13.3-p13.2
14	*HLA-DQB2*	*Major histocompatibility complex, class II, DQ beta 2*	1.23E-14	0.23	6p21
15	*LIPF*	*Lipase, gastric*	1.38E-14	16.59	10q23.31
16	*TOX3^a^*	*TOX high mobility group box family member 3*	1.45E-14	21.51	16q12.1
17	*ANXA10*	*Annexin A10*	1.50E-14	49.69	4q33
18	*VCAN*	*Versican*	1.82E-14	9.12	5q14.3
19	*ATP2A3a*	*ATPase, Ca++ transporting, ubiquitous*	1.92E-14	12.91	17p13.3
20	*GALNT10*	*Polypeptide N-acetylgalactosaminyltransferase 10*	1.98E-14	5.83	5q33.2
21	*GATA6*	*GATA binding protein 6*	2.00E-14	33.17	18q11.1-q11.2
22	*SPINK1*	*Serine peptidase inhibitor, Kazal type 1*	2.14E-14	58.02	5q32
23	*IGHA1*	*Immunoglobulin Heavy Constant Alpha 1*	2.73E-14	38.01	14q32.33
24	*PROM1*	*Prominin 1*	2.77E-14	26.19	4p15.32
25	*C6orf124*	*Chromosome 6 open reading frame 124*	2.97E-14	0.50	6q27
26	*ENAH*	*Enabled Homolog (Drosophila)*	3.30E-14	3.54	1q32.2
27	*FNDC3B*	*Fibronectin type III domain containing 3B*	3.38E-14	5.95	3q26.31
28	*VILL*	*Villin-like*	3.83E-14	10.06	3p21.3
29	*ADAM28^a^*	*ADAM metallopeptidase domain 28*	4.32E-14	8.44	8p21.2
30	*UGT2B15*	*UDP glucuronosyltransferase 2 family, polypeptide B15*	4.57E-14	18.60	4q13
31	*PLVAP*	*Plasmalemma vesicle associated protein*	4.77E-14	3.48	19p13.2
32	*ITPR3*	*Inositol 1,4,5-trisphosphate receptor, type 3*	4.96E-14	3.21	6p21
33	*IGLL5*	*IGLL5 immunoglobulin lambda-like polypeptide 5*	5.38E-14	25.73	22q11.22
34	*KCTD14*	*Potassium channel tetramerisation domain containing 14*	6.09E-14	9.05	11q14.1
35	*TFF1*	*Trefoil factor 1*	6.17E-14	40.23	21q22.3
36	*MUC6*	*Mucin 6*	6.26E-14	10.16	11p15.5
37	*GALNT6*	*Polypeptide N-acetylgalactosaminyltransferase 6*	6.38E-14	10.69	12q13
38	*FCER1A*	*Fc fragment of IgE, high affinity I, receptor; α polypeptide*	8.17E-14	0.14	1q23
39	*CD207*	*CD207 Molecule, Langerin*	8.44E-14	0.19	*2p13*
40	IGL@a	*Immunoglobulin lambda locus*	9.33E-14	19.78	22q11.2
41	*MLPH*	*Melanophilin*	9.97E-14	12.26	2q37.3
42	*PLAUR*	*Plasminogen activator, urokinase receptor*	1.17E-13	3.82	19q13
43	*IGKC*	*Immunoglobulin kappa constant*	1.19E-13	19.90	2p12
44	*PLA2G10*	*Phospholipase A2, group X*	1.20E-13	12.41	16p13.1-p12
45	*CTSE*	*Cathepsin E*	1.23E-13	39.20	1q31
46	*PRSS23*	*Protease, Serine, 23*	1.28E-13	5.52	11q14.2
47	*TMPRSS3*	*Transmembrane protease, serine 3*	1.29E-13	8.64	21q22.3
48	*GOLM1*	*Golgi membrane protein 1*	1.34E-13	31.45	9q21.33
49	*TRIB2*	*Tribbles Pseudokinase 2*	1.35E-13	3.77	2025.1
50	*KIAA0746*	*LOC285508*	1.41E-13	9.06	4p15.2

NOTE: Genes are listed in descending order by *P*-value. For genes with >1^a^ or >2^b^ probesets differentially expressed at *P*<1.12E-06 only the most significant probeset for the gene is shown.

**Table 3:** Summary showing top 50 differentially expressed genes in BE vs NC (the complete list is reported in Table S5).

No	Gene Symbol	Gene Name	*P-value*	Fold Change	Chromosomal Location
1	*DGKA*	*Diacylglycerol kinase, alpha 80 kDa*	6.74E-14	3.50	12q13.3
2	*SULT2A1*	*Sulfotransferase family, cytosolic, 2A*	2.41E-13	0.34	19q13.3
3	*HOXC6*	*Homeobox C6*	4.00E-13	5.56	12q13.3
4	*S100A10*	*S100 calcium binding protein A10*	8.86E-13	4.20	1q21
5	*PAK3*	*p21 protein (Cdc42/Rac)-activated kinase 3*	1.05E-12	0.40	Xq23
6	*RGS7*	*Regulator of G-protein signaling 7*	2.55E-12	0.44	1q23.1
7	*PDZK1IP1*	*PDZK1 interacting protein 1*	3.07E-12	4.21	1p33
8	*RAB11FIP2^a^*	*RAB11 family interacting protein 2 (class I)*	5.80E-12	0.24	10q26.11
9	*CEACAM6^a^*	*Carcinoembryonic antigen-related cell adhesion molecule 6*	6.60E-12	21.37	19q13.2
10	*MALL*	*Mal, T-cell differentiation protein-like*	7.17E-12	14.41	2q13
11	*FUT6*	*Fucosyltransferase 6*	8.26E-12	4.21	19p13.3
12	*MYRIP*	*Myosin VIIA and Rab interacting protein*	9.44E-12	0.20	3p22.1
13	*GABARAPL1^b^*	*GABA(A) receptor-associated protein like 1*	9.75E-12	0.48	12p13.2
14	*ESRRG*	*Estrogen-related receptor gamma*	1.05E-11	0.06	1q41
15	*CLDN7*	*Claudin 7*	1.51E-11	8.37	17p13.1
16	*KCNE2*	*Potassium voltage-gated channel, Isk-related family, member 2*	1.59E-11	0.06	21q22.12
17	*C3orf18*	*Chromosome 3 open reading frame 18*	1.71E-11	0.48	3p21.3
18	*DDB2*	*Damage-specific DNA binding protein 2, 48 kDa*	1.74E-11	2.13	11p12-p11
19	*FUT3*	*Fucosyltransferase 3*	2.13E-11	3.98	19p13.3
20	*AGFG2*	*ArfGAP with FG repeats 2*	2.14E-11	2.15	7q22.1
21	*SH3GL2*	*SH3-domain GRB2-like 2*	2.16E-11	0.14	18q21.3
22	*PFTK1*	*Cyclin-dependent kinase 14*	2.42E-11	0.33	7q21-q22
23	*PCSK5*	*Proprotein convertase subtilisin/kexin type 5*	2.76E-11	4.41	9q21.3
24	*STX12^a^*	*Syntaxin 12*	2.76E-11	0.46	1p35.3
25	*SERPINB5*	*Serpin peptidase inhibitor, clade B (ovalbumin), member 5*	2.84E-11	21.98	18q21.3
26	*CLDN4*	*Claudin 4*	3.57E-11	4.91	7q11.23
27	*TM4SF1*	*Transmembrane 4 L six family member 1*	3.71E-11	4.80	3q21-q25
28	*RNF19B*	*Ring finger protein 19B*	3.72E-11	2.21	1p35.1
29	*AGXT2L1*	*Alanine-glyoxylate aminotransferase 2-like 1*	4.07E-11	0.06	4q25
30	*PTGER3*	*Prostaglandin E receptor 3*	4.86E-11	0.14	1p31.2
31	*CEACAM1*	*Carcinoembryonic Antigen-Related Cell Adhesion Molecule 1*	5.57E-11	3.60	19q13.2
32	*ANXA1*	*Annexin A1*	6.04E-11	14.57	9q21.13
33	*ANXA2^a^*	*Annexin A2*	6.10E-11	2.78	15q22.2
34	*CCPG1b*	*Cholecystokinin B receptor*	6.13E-11	0.33	15q21.1
35	*APOBEC2*	*Apolipoprotein B mRNA editing enzyme, catalytic polypeptide-like 2*	6.29E-11	0.24	6p21
36	*MYOF*	*Myoferlin*	6.34E-11	3.50	10q24
37	*NNT*	*Nicotinamide Nucleotide Transhydrogenase*	6.37E-11	0.40	5p12
38	*S100A14*	*S100 calcium binding protein A14*	7.06E-11	5.48	1q21.3
39	*KCNJ16*	*Potassium inwardly-rectifying channel, subfamily J, member 16*	7.11E-11	0.05	17q24.3
40	*SLC25A4*	*SH3-domain GRB2-like 2*	7.54E-11	0.39	9p22
41	*ALDH6A1b*	*Aldehyde dehydrogenase 6 family, member A1*	7.87E-11	0.39	14q24.3
42	*CD9*	*Cell cycle progression 1*	8.04E-11	4.35	12p13.3
43	*CYFIP2*	*Cytoplasmic FMR1 interacting protein 2*	8.12E-11	0.24	5q33.3
44	*TP53I3*	*Tumor protein p53 inducible protein 3*	8.68E-11	3.81	2p23.3
45	*INPP1*	*Inositol polyphosphate-1-phosphatase*	8.79E-11	3.05	2q32
46	*GJB3*	*Gap junction protein, beta 3, 31 kDa*	8.97E-11	3.12	1p34
47	*APLP1*	*Amyloid beta (A4) precursor-like protein 1*	8.98E-11	0.15	19q13.1
48	*Hs.677385*	*cDNA clone LNG01731*	9.48E-11	0.26	7p13-p12
49	*CDH2*	*CD9 molecule*	9.73E-11	0.20	18q11.2
50	*ANXA2P2*	*Annexin A2 Pseudogene 2*	9.77E-11	2.67	9p13.3

NOTE: Genes are listed in descending order by *P*-value. For genes with >1^a^ or >2^b^ probesets differentially expressed at *P*<1.12E-06 only the most significant probeset for the gene is shown.

Between BE vs NE tissues, *mucin 5AC (MUC5AC)*, *carbonicanhydrase II (CA2)*, and *claudin 18 (CLDN18)* genes had the most significant differentially-expressed levels (*P*<1.5E-15, Table 2), while *MUC5AC* (69 fc), *serine peptidase inhibitor, Kazal type 1* (*SPINK1*, 58 fc), and *CLDN18* (54 fc) had the greatest fold-change in expression levels (Table 2 and Table S4). Other significantly differentially-expressed genes in BE vs NE included *gastrokine 1* (*GKN1*, 34 fc, *P* = 2.72E-15), *TFF2* (37 fc, *P* = 5.47E-15), *SULT1C2* (24 fc, *P* = 4.86E-15) *prominin 1* (*PROM1*, 27 fc, *P* = 7.10E-16), and *trefoil factor 1* (*TFF1*, 40.0 fc, *P* = 6.17E-14) (Table 2). Likewise, statistical significance was greatest in the BE vs NC tissue expression comparisons for *diacylglycerol kinase, alpha 80 kDa (DGKA), sulfotransferase family cytosolic, 2A (SULT2A1)*, *homobox C6 (HOXC6), S100 calcium binding protein A10 (S100A10)* (all *P*<1.0E-12) (Table 3); while the fold changes of the highest magnitude were observed for *ATPase, H+/K+ exchanging, beta polypeptide* (*ATP4B*, 0.04 fc); *chitinase, acidic* (*CHIA*, 0.04 fc); and keratin 13 (*KRT13*, 31.0 fc) (Table S5). Other significantly differentially-expressed genes in BE vs NC tissues included P21 protein-activated kinase 3 (*PAK3*, 0.4 fc, *P* = 1.05E-12), *regulator of G-protein signaling 7 (RGS7)* (*IGKC*, 20 fc, *P* = 1.19E-13), and *carcinoembryonic antigen-related cell adhesion molecule 6* (*CEACAM6*, 21 fc, *P* = 6.64E-12) (Table S5).

### Validation of microarray results by qRT-PCR

We validated the microarray expression of nine genes from matched BE, NE, and NC tissues using TaqMan qRT-PCR. Six of these genes, which were validated in independent tissue triplets (120 samples total) in the current study, have previously been reported in the development of BE (Table S6). In summary, validations were in agreement with microarray results and details of probes, kits, tissue numbers and significant expression levels are presented in Table S6.

### Pathway and regulatory network analysis of the common differentially expressed genes in BE vs NE and BE vs NC

**Figure 3:**
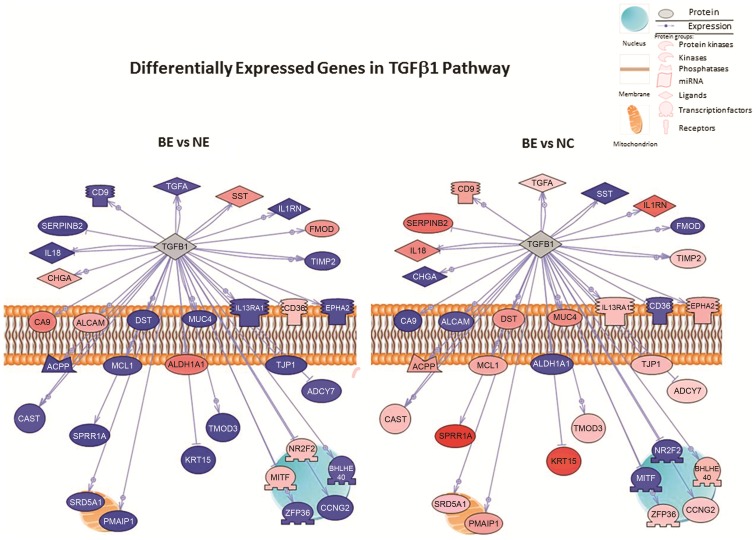
Pathway-based analyses of common differentially expressed genes in BE vs NE and BE vs NC comparisons. Two hundred and five overlapping genes were identified between BE vs. NE and BE vs. NC tissues after Bonferroni correction (*P*<1.12-E06) and with a 2-fold or greater differential expression. Diagram illustrates the TGF-β1 signaling pathway in BE vs NE and BE vs NC comparative groups. The direction and/or magnitude of expression is inverse for thirty two genes in the TGF-β1 network between the comparative groups. Genes include: *SERPINB2, TMOD3, EPHA2, KRT15, ADCY7, SST, FMOD, CD36, CA9, ALCAM, IL1RN, TIMP2, CAST, MITF, SPRR1A, BHLHE40, MCL1, IL13RA1, ACPP, SRD5A1, NR2F2, ALDH1A1, DST, TJP1, CD9, TGFA, MUC4, PMAIP1, ZFP36, CCNG2, IL18* and *CHGA*. Data source: Signaling Pathways, Ariadne Pathways. Primary red and blue colors and shading indicate the direction and degree of differential expression with pink to red indicating degrees of increasing upregulation and light blue to darker blue indicating increasing downregulation. Grey indicates a gene product that is part of the pathway, but is absent in the experimental list tested. Abbreviations: BE, Barrett's; NE, normal squamous epithelium; NC, normal gastric cardia epithelium.

We determined the overlap of differentially expressed genes between the BE vs NE and BE vs NC sets and identified 205 genes (Figure 2 and Table S7). We then used Pathway Studio 9.0 to determine the functional groups, processes, upstream regulators, and pathways that were overrepresented in this common gene set. A number of functional categories were found in the 205 genes, including genes encoding nucleotide binding proteins, hydrolase activity, and GTP binding (Table S8). Evaluation of biological processes related to the 205 differentially expressed genes indicated that the greatest number of gene subsets were involved in response to drugs, cell adhesion, small GTPase-mediated signaling and lipid metabolic processes (Figure 2 and Table S8). However, the proportion of genes identified in relation to the total number of genes involved in each process according to the database was greatest for cellular response to insulin stimulus, followed by epidermis development, and negative regulation of endopeptidase activity. Analyses of the 205 genes revealed numerous relationships that could be mediated through a number of key upstream regulatory proteins, including TGF-β, IL1B, TP53, and INS (Figure 3 and Table S8); the direction and/or magnitude of expression were not the same for a number of genes in the specific networks between the BE vs NE and the BE vs NC comparative groups (Figure 3). We also aimed to determine if the 205 common differentially expressed genes correlated with specific signaling pathways, and found that the greatest number of these genes (n = 67) mapped to the Atlas of Signaling pathway, a single overview pathway depicting the main cellular signal transduction channels (from receptors to transcription factors). Other common pathways included cell cycle regulation (n = 24 genes), Notch (n = 19 genes), and the guanylate cyclase pathway (n = 18 genes); these latter two pathways represent those containing the greatest proportion of - genes (Table S8) relative to the total number of genes in the respective pathway (Pathway Studio 9.0). The top ten hits for all pathway-based analyses are shown in Table S8.

## Discussion

There is no accepted hypothesis for the molecular mechanism underlying the development of BE. However, identifying genes that are differentially expressed in BE compared to normal esophageal (NE) squamous epithelium and normal gastric cardia (NC) epithelium should improve our understanding of the biology of BE development and may also identify genes whose expression may be useful in the diagnosis and clinical management of BE. The results of the present study extend previous findings indicating that BE shares phenotypic elements with normal epithelia from both the squamous esophagus and the gastric cardia [19], [20]. Importantly, the analyses of matched tissue samples from a large number of BE patients using a high-density microarray allowed us to evaluate the expression levels of 14,500 well-annotated genes in BE, NE, and NC tissues from the same patients.

In relation to individual gene expression changes between paired BE vs NE, and BE vs NC tissues from BE patients, we identified more significant genes than have been reported previously [21], [22]. PCA analysis of these dysregulated genes suggests that there are more similarities in gene expression profiles between BE and NC than between BE and NE.

In support of previous data, we identified many genes previously reported to be associated with BE metaplasia. For example, in BE vs NE, intestinal markers such as trefoil factors (TFF) 1, 2, and 3 were upregulated as were mucins [23], [25], particularly *MUC5AC*, which has been associated with wound healing. We observed increased expression of lysozyme, a potent non-immunological antibacterial enzyme previously shown to be upregulated in BE [30]. We also observed increased expression of *CLDN18* and *CLDN10* in BE vs NE [26]. Similarly, in BE vs NC, the expression of claudin and mucin genes were altered. In agreement with other data [21]–[26], [31], keratin expression profiles revealed numerous changes in BE metaplasia compared to NE (e.g., *KRTs 4*, *8*, *15*, and *20*) and NC (*KRTs 5*, *6*, *13*, *14*, and *15*) tissues.

Current evidence suggests the conversion of NE to BE metaplasia can arise from four potential mechanisms. The first mechanism is transdifferentiation which involves the irreversible switch of mature squamous esophageal epithelial cells to another differentiated cell [25], [32]. The intestinal epithelial-associated claudel-type homeobox (CDX) transcription factors *CDX1* and *CDX2* have been implicated in the pathogenesis of BE and in the transdifferentiation of stratified squamous epithelia into columnar intestinal epithelia [25]. In the present study, we did not observe significant expression changes in *CDX2* mRNA in either BE vs NE (1.1 fc, P = 0.36) or in BE vs NC (1.1 fc, P = 0.90) tissue comparisons. In addition, at the individual or BE patient level, only 11 of 40 BE tissue comparisons had a ≥1.5 fc increase in *CDX2* mRNA compared to matched NE tissues. While data from smaller qRT-PCR studies [33], [34] previously suggested an increase in *CDX2* mRNA in BE, current data from array profiling suggests that *CDX2* mRNA is not dramatically upregulated in BE vs NE [23]–[25], [31], [35], but paradoxically, CDX2 protein is overexpressed in most BEs [36], [37]. However, we did observe a significant overexpression of *CDX1* mRNA in BE vs NE (1.38 fc, P = 0.008) and BE vs NC (1.28 fc, P = 0.02) comparisons as well as other more significant classical gene markers of BE (e.g., *Villin*, *MUC2*, *MUC5B*, *KRT20*, and *CLDN18*) [25], [26], [35]. A second mechanism [38] for the development of BE involves opportunistic cell lineage, whereby unique embryonic progenitor cells existing in the squamocolumnar junction physically migrate to replace damaged p63-deficient squamous cells in the adult esophagus. Several studies have shown that p63 expression, which is critical for the development and differentiation of normal esophageal epithelia [39], is lost in BE compared to NE [40]. We also observed significantly decreased expression of *p63* in BE vs NE, while *p63* expression was significantly increased in BE vs NC. Interestingly, Wang et al. [38] also reported that *CDX2* was not upregulated in murine esophageal cells lacking p63, in spite of the columnar phenotype of the cells. More recent evidence suggests a third and potential epigenetic mechanism which may be at work in the development of BE and involves alteration of *HOX* gene expression [41]. In agreement with di Pietro et al. [41], we also observed significant expression changes in the *HOX* genes in BE vs NE and BE vs NC. In particular, we observed increased expression of *HOXB5*, *HOXB6*, and *HOXB7* and significant activation of the downstream intestinal markers *KRT8*, *KRT18*, and *KRT20* in BE vs NE.

The final proposed mechanism suggests that BE may develop from the conversion of a tissue-specific stem or pluripotential cell in the esophagus (e.g., a bone marrow-derived pluripotential stem cell), which has the capacity for unlimited or prolonged self-renewal [42]. We detected significantly increased expression of *prominin-1* (*PROM1*) in BE vs NE but not in BE vs NC. *PROM1* (also known as *CD133*) is a suggested marker for intestinal stem cells that are susceptible to neoplastic transformation and is recognized as a stem cell marker in several tissues and in many cancers [43]. The sex-determining region Y (*Sry*) box-containing (SOX) factors are a family of transcription factors that are emerging as potent regulators of stem cell maintenance and cell fate decisions in multiple organ systems [44]. While SOX2 is essential for the maintenance of embryonic stem cells [44], [45] its expression is also essential for the normal development of the NE [46]. Also, expression of SOX9 in NE cells is sufficient to drive columnar differentiation of squamous epithelium and expression of an intestinal differentiation marker, reminiscent of BE [47]. An increased expression of SOX9 and SOX2 protein has also been described for EA tumor cell lines compared to BE cells [45]. We found that *SOX9* (3.37 fc and P = 4.05E-12) and *SOX4* mRNAs (2.88 fc and P = 3.89E-11) were upregulated, while *SOX2* (0.46 fc and P = 9.36E-07) and *SOX15* mRNAs (0.34 fc and P = 3.12E-09) were significantly downregulated in BE tissues compared to NE, but not in BE vs NC. Interestingly, downregulation of SOX2 can lead to an intestinal phenotype in gastric epithelial cells via downregulation of *MUC* and *CDX* expression [48].

Besides individual genes in each tissue comparison, we also determined which genes were dysregulated in both tissue comparison groups. These 205 common differentially expressed genes were overrepresented by genes involved with nucleotide binding and GTP binding/activity as well as peptidase inhibitor activity. Precursors of pepsinogen A and pepsinogen C (progastricsin) have been demonstrated in BE epithelium [49]. We observed an 18-fold upregulation of pepsinogen C in BE vs NE and a 5.3 fold downregulation in BE vs NC, results that may reflect the prevalence of GERD (64%) in the BE patients in the study. An overrepresentation of peptidase genes in BE compared to NE (and in EA compared to NE) was previously reported by Greenawalt *et al.* [23]. Recent but limited data suggests that the involvement of insulin signaling may be important for BE development, particularly in the progression from BE to EA via increased expression of insulin-like growth factor 1 receptor (IGF1R) [50]. While we did not observe increased expression of IGFR1 in either tissue comparison in this study, cellular response to insulin showed the greatest enrichment of differentially-expressed genes; further, we observed a significant upregulation of insulin receptor mRNA levels in BE vs NE (4.6 fc) and BE vs NC (1.8 fc).

Performing pathway-based analysis of the expression of the 205 genes commonly dysregulated in both comparison groups allowed us to evaluate relationships between genes and their encoding proteins as well as the associated pathways in which these proteins are involved. Analyses of the 205 genes differentially expressed in both comparison groups and potential upstream regulators revealed connectivity between many of the genes associated with BE status. However, for the majority of the gene relationships this connectivity appeared to be mediated through a number of key upstream regulatory factors that included TGF-β1 and INS. Interestingly, the direction of differential expression of each gene downstream of the regulator was different between the two comparison groups and in some cases was the inverse (Figure 3). In particular, more genes were downregulated in the TGF-β1 network in BE vs NE compared to BE vs NC (Figure 3), suggesting a loss of TGF-β signaling in the former. Several investigators have reported impaired TGF-β signaling in the BE metaplasia-dysplasia-adenocarincoma sequence [5], [45], [51]. Mendelson *et al.* [45] recently evaluated TGF-β and Notch signaling in NE and BE tissues using immunohistochemistry. They found further evidence of loss of TGF-β in BE (and BE-associated EA) as well as activation of Notch in BE-associated EA compared to normal squamous epithelium as characterized by increased expression of HES-1 and JAG1 proteins. We also observed enrichment of 19 genes in the Notch signaling pathway (Table S8). The majority of Notch pathway genes (23 of 32) were downregulated in BE vs NE. Previous evidence suggests that in esophageal squamous cells, Notch signaling is growth repressive [52]. Also, in contrast, 23 of 32 Notch-associated genes were significantly upregulated in BE vs NC. Thus, in agreement with other studies, the present results suggest that a loss of TGF-β signaling as well as Notch signaling may be important in the development of BE metaplasia, possibly by disrupting the ability of cells to differentiate or maintain the differentiated state. However, depending on whether BE is compared to NE or NC, the direction and/or magnitude of disruption in these pathways may appear very different, a finding which may have implications for targeted therapy for BE metaplasia.

The use of whole tissue biopsies is a limitation in this study, as an admixture of epithelium with inflammatory and stromal cells could affect the genes identified. However, stromal cells can have a significant impact on adjacent epithelia and, considering the implicated role of TGF-β signaling in the development of BE, epithelial-mesenchymal interactions are likely to be an important contributory factor to the development of BE [24], [53].

In conclusion, the results of the present study extend previous findings indicating that BE shares phenotypic elements with normal epithelia of the squamous esophagus (NE) and the gastric cardia (NC). The analyses of BE, NE, and NC tissues from the same patient provides a robust picture of differential gene expression of BE compared to other studies. The results of this study provide a rich source of data for the analysis of specific genes and pathways in relation to the development of BE metaplasia and for the identification of potential new biomarkers and/or treatment targets.

## Supporting Information

Table S1 **Demographic and risk factor information for BE cases.**
(XLSX)

Table S2 **Endoscopy findings for BE cases.**
(XLSX)

Table S3 **Pathology findings for cardia, BE, and squamous esophagus biopsies from BE cases.**
(XLSX)

Table S4 **Summary of 1645 probe sets in BE versus NE (P<1.12E-06 with 2-fold or greater change).**
(XLSX)

Table S5 **Summary of 782 probe sets in BE versus NC (P<1.12E-06 with 2-fold or greater change).**
(XLSX)

Table S6 **Microarray-based and qRT-PCR-based expression results for genes selected for validation in discovery samples and independent replication in new samples.**
(XLSX)

Table S7 **Common genes significantly differentially expressed (P<1.12E-06 with 2-fold or greater change in expression) in BE vs NE and BE vs NC tissues comparisons.**
(XLSX)

Table S8 **Gene function and pathway-based analyses of common genes significantly differentially expressed in BE vs NE and BE vs NC tissues comparisons.**
(XLSX)
